# Multipartite viruses: adaptive trick or evolutionary treat?

**DOI:** 10.1038/s41540-017-0035-y

**Published:** 2017-11-09

**Authors:** Adriana Lucía-Sanz, Susanna Manrubia

**Affiliations:** Grupo Interdisciplinar de Sistemas Complejos (GISC), National Centre for Biotechnology (CSIC), c/Darwin 3, 28049 Madrid, Spain

## Abstract

Multipartitism counts amongst the weirdest lifestyles found in the virosphere. Multipartite viruses have genomes segmented in pieces enclosed in different capsids that are independently transmitted. Since all segments have to meet in the host for complementation and completion of the viral cycle, multipartite viruses are bound to fight the loss of genomic information. While this is an obvious disadvantage of this strategy, no consensus on its actual advantages has been reached. In this review we present an exhaustive summary of all multipartite viruses described to date. Based on evidence, we discuss possible mechanistic and evolutionary origins of different groups, as well as their mutual relationships. We argue that the ubiquitous interactions of viruses with other unrelated viruses and with subviral elements might be regarded as a plausible first step towards multipartitism. In agreement with the view of the Virosphere as a deeply entangled network of gene sharing, we contend that the power of multipartitism relies on its dynamical and opportunistic nature, because it enables immediate adaptive responses to environmental changes. As such, perhaps the reasons for its success should be shought in multipartitism itself as an adaptive mechanism, to which its evolutionarily short-lived products (that is, the extant ensemble of multipartite viral species) are subordinated. We close by discussing how our understanding of multipartitism would improve by using concepts and tools from systems biology.

## Viruses with multipartite genomes: unsolved puzzles on their origins and advantages

The faithful transmission of the genome of an organism is a fundamental step to preserve information essential for survivability. However, multipartite viruses thrive with segmented genomes that propagate in independent viral particles. Though this adaptive strategy appears as counterintuitive and suboptimal, multipartitism is common in the viral world and has very likely arisen several times. Here we review the distribution and abundance of multipartite viruses and discuss possible evolutionary pathways for their emergence. Though no clear advantage of multipartitism has been identified, we suggest that the high prevalence of this strategy relies on its dynamic and opportunistic nature, and that it can only be understood in an ecological context. A systems biology perspective could help understanding some of the open questions regarding this weird lifestyle, while multipartitism could in turn inspire design principles based on the simultaneous exploration of an exploding number of transient collaborative associations.

## Introduction

We are all acquainted with viruses. Often as a result of experience, we know how pathogenic they can be and fear the effects of viral infections. We are told that viruses have found ways to infect all cellular life forms, and that they mutate and adapt fast enough to be extremely hard to control or eradicate. Since the discovery of Tobacco mosaic virus (TMV) at the end of the XIX century,^1^ Virology has been strongly conditioned by the research of those viruses with pernicious effects on crops, livestock or humans, resulting in a generalized appreciation of viruses as independent, opportunistic and greedy parasites. However, this view is gradually changing. The effects viruses cause in an infected host are diverse and on many occasions innocuous.^2^ Actually, the more we know about viruses, the more we see them departing from the picture of a simple pathogenic agent. The last two or three decades have witnessed important conceptual changes in our understanding of virus-like elements,^3^ as well as fresh attempts to survey viral diversity in a balanced manner.^4^ Our skewed view of the Virosphere could soon change if viruses identified through metagenomic techniques are incorporated into current catalogs.^5^ Though we seem to know the mechanistic principles that endow viruses with their adaptive success, there are major unknowns from the viewpoint of their adaptive strategies and their lifecycles. In agreement with their complex biology, viruses also present intricate lifestyles. Following the principle of the evolutionary tinkerer, some virus genes are related to cellular genomes and different mobile replicating elements,^6^ and the other way round.^7^ We can only make full sense of viruses and viral lifestyles if we leave aside their study as independent pathogens and integrate them in a dynamical scenario where gene exchange, functional reuse and more-than-fuzzy distinctions among “viral species” (and other selfish genomic elements^8^) become the norm.

This review deals with a particular class of viruses known as multipartite viruses, first described in the 1960 decade,^9,10^ In the literature, multipartite viruses have been also called coviruses, multicomponent, multiparticle or multicompartment viruses. Here, we are considering three main types of viral genome configurations that will be termed non-segmented, segmented and multipartite. Non-segmented and segmented viruses transport all the genetic material needed to complete the viral cycle inside a unique viral particle; multipartite viruses have by definition segmented genomes, and their chromosomes are distributed among two or more virus particles. Co-infection is therefore a requirement for survivability of this latter type. There are many more open questions than certainties in our understanding of multipartite viruses.^11–13^ Among all, the main puzzle is why multipartite viruses do exist at all.^14^ Though mostly limited to plants, multipartition may have appeared repeatedly in evolution. Certainly, multipartitism is not anecdotal, neither a frozen evolutionary accident nor a serendipitous discovery. Despite our current lack of knowledge on the mechanisms that may endow multipartite viruses with an adaptive advantage that compensates for their apparently weird and costly lifestyle, there is no alternative but beginning by assuming that multipartitism is a stable evolutionary strategy, *sensu* Maynard-Smith.^15^

We start by summarizing known (and some new) facts on the biology of multipartite virus. For further details on the molecular biology of multipartitism the reader is referred to recent reviews that have tackled this issue in depth.^12,16^ Our main goal here is to discuss possible scenarios for the origin and evolution of multipartitism as an evolutionary strategy, and highlight perspectives for its study. From a systems biology viewpoint, multipartite viruses could be better depicted as complex ensembles of genes that, perhaps transiently, associate in a cooperative fashion to yield a variety of viral species.^17^ We contend that multipartitism can only be understood in an ecological context, that is when the host and the epidemiology of multipartite viruses—and other possible associated selection pressures—are taken into account. Comparing multipartitism and alternative genomic architectures, as well as exploring the environments where multipartitism occurs might pave the way towards disentangling the actual costs and advantages of this as of today puzzling lifestyle.

## Prevalence and organization of segmented and multipartite viruses

**Fig. 1:**
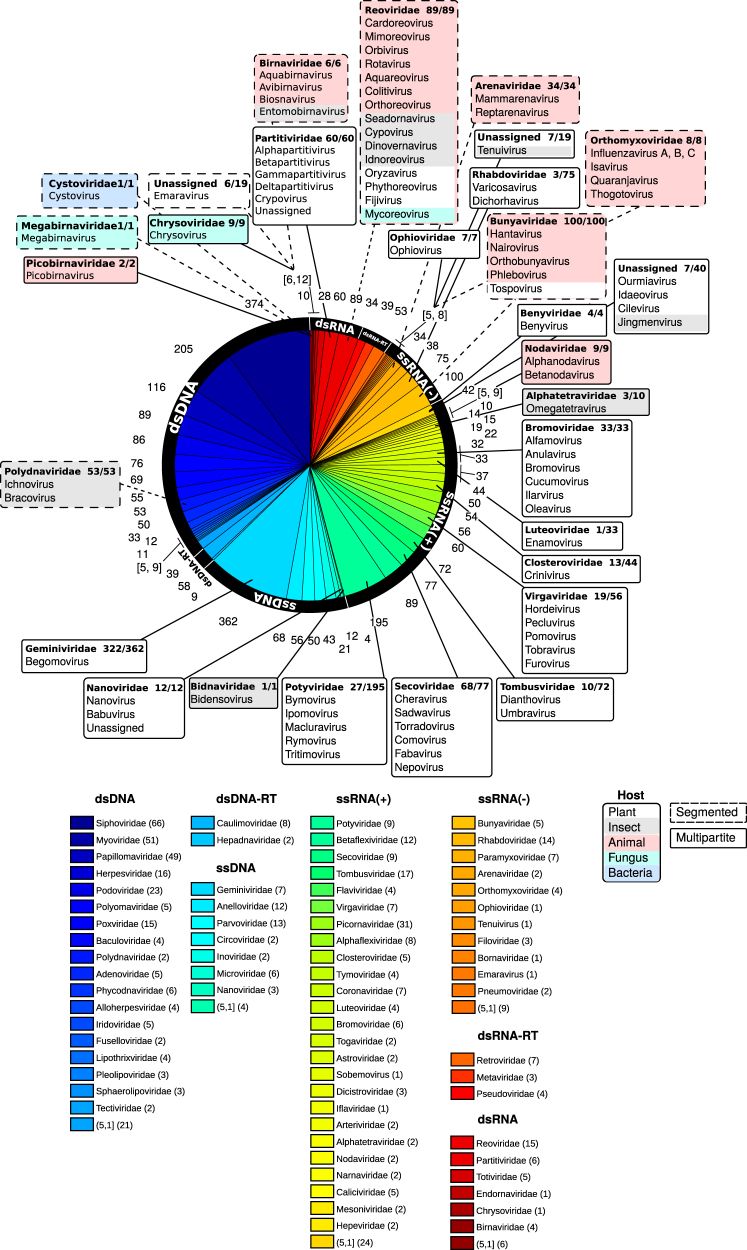
Pie chart showing the abundances of all currently annotated species in the ICTV (2015) for each viral family.^18^ Figures around the pie indicate the number of species in a given family. Colors link to a viral family in the color legend. In the legend the number of genera corresponding to each family is given in parenthesis. Families with four or less species are merged together. Pop charts contain multipartite (solid line) and segmented (dashed line) viral families (name in bold face), and show the number of multipartite or segmented species/total number of species. Background colors of the pop charts indicate the main host. List of families with four or less viral species, with the number of genera first given between brackets if different from one: *ssDNA: Bacilladnavirus 1, Spiraviridae 1, Genomoviridae 1, Bidnaviridae 1; *ssRNA(+): Leviviridae (2) 4, Hypoviridae 4, Benyviridae 4, Ourmiavirus 3, Bacillarnavirus 3, Albetovirus 3, Sinaivirus 2, Jingmenvirus 2, Permutotetraviridae 2, Sarthroviridae 1, Carmotetraviridae 1, Barnaviridae 1, Alvernaviridae 1, Gammaflexiviridae 1, Marnaviridae 1, Roniviridae 1, Virtovirus 1, Polemovirus 1, Papanivirus 1, Labyrnavirus 1, Idaeovirus 1, Higrevirus 1, Cilevirus 1, Aumaivirus 1; *ssRNA(−): Nyamiviridae (2) 4, Deltavirus 1, Wastrivirus 1, Crustavirus 1, Chengtivirus 1, Arlivirus 1, Anphevirus 1, Sunviridae 1, Mymonaviridae 1; *dsRNA: Amalgaviridae 4, Picobirnaviridae 2, Botybirnavirus 1, Quadriviridae 1, Megabirnaviridae 1, Cystoviridae 1

**Fig. 2:**
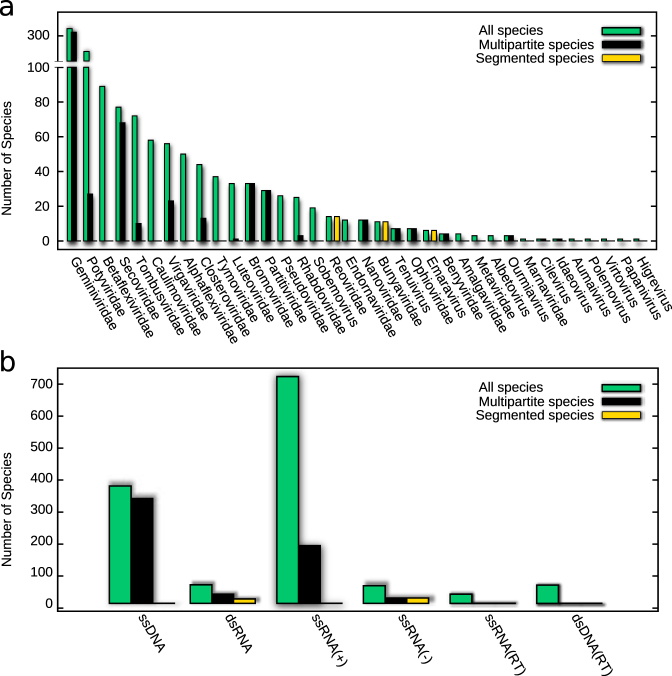
Histogram of the number of plant virus species **a** per viral family and **b** per genome type. Green bins represent the total number of plant virus species; black bins stand for the number of multipartite species and yellow bins for the number of segmented species

Most of our current knowledge on the prevalence of multipartite viruses is recorded on publicly available databases from the International Committee on Taxonomy of Viruses (ICTV)^18^ and ViralZone.^19^ Figure 1 summarizes the distribution of multipartite and segmented viral species within the Virosphere, and highlights the heterogeneous distribution of genome types and hosts. About 17% of all annotated viral species have a multipartite genome, while 9% are segmented species (8 and 5% of the genera respectively, according to data from 2015). Multipartite viruses infect mostly plants (90% of species and genera), being present in 13 out of 24 plant viral families (Fig. 2a). As a plant pathogen, they display circular ssDNA, dsRNA or ssRNA of positive and negative polarity genomes (Fig. 2b). Most multipartite viruses (90% of species and 60% of genera) are transmitted by an animal vector, like aphids, whiteflies, planthoppers, mites or nematodes, in a non-circulative, circulative or propagative manner.^20^ A small amount of genera are transmitted by plant pathogens such as fungi or protozoans (5–6%). The rest, about 35% of the genera, are vertically transmitted plant viruses. Note that this transmission strategy is free from the cost of a high multiplicity of infection (MOI) required for horizontally transmitted multipartite viral forms to succeed. Amongst 18 proposed multipartite families, only four infect animals exclusively: Bidnaviridae,^21,22^ Alphatetraviridae^23^ (both infecting insects), Nodaviridae (infecting fishes),^24^ and a possible bipartite virus infecting mammals from the family Picobirnaviridae.^25^ Figure 2 represents the abundances of plant virus species per genome type and viral family. In some cases, like for Begomovirus, the number is inflated due to their very high recombination rate, while other families might be underrepresented at the species level.

Table 1 lists all proposed families of multipartite viruses and their capsid structure, their abundances in terms of genera and species, the number of genomic segments and the hosts they infect. Looking at the numbers, there is an overwhelming majority of bipartite species, followed by a significant number of species with three or four segments. The number of species with over four segments is rare, with the exception of the quite diverse Nanoviridae family. Most genera infecting hosts different from plants are bi or tripartite, with some exceptions containing a few species. For example, Tenuivirus have four to six segments and infect planthoppers^26^ apart from plants, and Jingmenvirus infect only animals.^27^ Other examples will be discussed later.

**Table 1:** Summary of viral families with multipartite genomes

Capsid	Family (Genera)	Segments	Species	Host
ssDNA				
	*Bidnaviridae* (1)	2	1	Insect
	*Nanoviridae* (3)	6−8	12	Plant
	*Geminivirade*	2 or 1 and satellite	322	Plant
ssRNA (+)				
	*Alphatetraviridae* (1)	3	3	Insect
	*Luteoviridae* (1)	2	1^a^	Plant
	*Nodaviridae* (2)	2−3	9	Animal
	*Secoviridae* (6)	2	68	Plant
	*Tombusviridae* (2)	2	10	Plant
	*Benyviridae* (1)	2, 4−5^b^	4	Plant
	*Virgaviridae* (6)	2 or 3^c^	23	Plant
	*Bromoviridae* (6)	3	33	Plant
	*Closteroviridae* (1)	2	13	Plant
	*Potyviridae* (5)	2	27	Plant
	Unassigned (4)^d^	2, 3, 3, 4	1, 1, 3, 2	Plant, Animal
ssRNA (-)				
	*Ophioviridae* (1)	3−4	7	Plant
	*Rhabdoviridae* (2)	2	3	Plant, Animal^e^
	Unassigned (1)^f^	4−6	7	Plant, Animal
dsRNA				
	*Chrysoviridae* (1)	4	9	Fungus
	*Partitiviridae* (6)	2^g^	60	Plant, Fungus, Protozoan
	*Picornabirnaviridae* (1)	2^h^	2	Animal

Columns display the genome type, the name of the family and the number of genera (in brackets), the number of genome segments, the number of described species, and the host

^a^ The unique species Pea enation mosaic virus is a bipartite virus whose segments belong to families Tombusviridae and Luteoviridae, each expressing their own RdRp

^b^ Benyviridae have 2 bipartite species and another 2 with 2 constitutive genes and a variable number of segments, up to 5, of function unknown^16^

^c^ Genera Hordeivirus, Pomovirus are tripartite, the rest are bipartite

^d^ Unassigned genera: Ourmiavirus (three species, three segments), Idaeovirus (one species, 2 segments), Cilevirus (3 species, 2 segments) and Jingmenvirus (two species, four segments)^27^

^e^ Genera *Varicosavirus* and *Dichorhavirus* are bipartite infecting plants. *Rhabdoviridae* non-segmented genera infect vertebrate and invertebrate animals.^106,107^ Bipartite *Rhabdoviridae* infect only plants

^f^ Genus *Tenuivirus* ^26^

^g^ *Partitiviridae* is a recently reestructured family which contains genera infecting only plants, only fungi, both simultaneously, and also one genera infecting protozoa. Segments are thought to be independently encapsidated^108^

^h^ *Picobirnaviridae* bipartite genome thought to be independently encapsidated^25^

*sat*: satellite. Animals include vertebrates and invertebrates; insects only invertebrates

Attending to genome composition, multipartite RNA plant viruses present two constitutive segments containing essential genes for infection, with extra segments with not well known functions usually needed to accomplish infection, such as in the families Benyviridae and Ophioviridae. The tripartite family Bromoviridae and genera Hordeivirus and Pomovirus from the family Virgaviridae are an exception to the former rule, since they bear constitutive genes distributed among all the three segments. Multipartite DNA plant viruses appear with two types of genome configurations. A representative of the first type is Begomovirus (a genus in the Geminiviridae family) which has a principal segment containing the main genes for infection. This segment is accompanied either by an auxiliary segment or by a satellite that modify host range and symptoms.^28^ The second type is represented by the Nanoviridae family. This is an extreme case of multipartitism, with each of the 6 to 8 separated segments coding for a gene—though not all segments are essential for infection in vitro.^29^

Segmented viruses (Table 2) display a broader host range and reach a higher number of segments. The majority are membrane-enveloped viruses, barring Birna, Megabirna, and Reoviridae families. Interestingly, viral families with segmented genomes only contain species with this architecture, while most families with multipartite genera also contain non-segmented genera. This suggests a higher plasticity of multipartite viral genomes, a fact indirectly supported by the rapidly growing number of multipartite species annotated from 1 year to another (48 new species of multipartite viruses from 2014 to 2015).^18^

**Table 2:** Summary of viral families with segmented genomes—in a single capsid

Family (Genera)	Segments	Species	Host
*dsDNA*			
*Polydnaviridae* (2)	10–11	53	Animal
*ssRNA (−)*			
*Arenaviridae* (2)	2	34	Animal
*Bunyaviridae* (5)	3	100	Plant, Animal
*Orthimyxoviridae* (6)	6 or 8	8	Animal
Unassigned (1)^a^	4	6	Plant
*dsRNA*			
*Birnaviridae* (4)^b^	2	6	Animal
*Cystoviridae* (1)	3	1	Bacteria
*Megabirnaviridae* (1)^b^	2	1	Fungus
*Reoviridae* (15)^b^	8-12	89	Animal, Plant, Fungus

Columns display the genome type, the name of the family and the number of genera (between brackets), the number of segments, the number of described species, and the host

^a^ Genus Emaravirus

^b^ Non-enveloped families

## On the possible origins of multipartitism

The viromes of prokaryotes and eukaryotes are qualitatively different, especially regarding genome type. While prokaryotes are infected predominantly by dsDNA viruses, the diversity of RNA viruses seems to have flourished in eukaryotic cells. Together with information on the origin of some important hallmark viral proteins, a picture of a coarse-grained evolutionary hierarchy for viral genomes begins to emerge. Multipartite virus, which infect eukaryotes exclusively, are suspected to have different evolutionary origins, mainly attending to their genome type. Whereas eukaryotic viruses of RNA genome seem to have a possible old origin in an ssRNA(+) ancestor, linked to eukaryogenesis,^30^ eukaryotic ssDNA viruses could have appeared later in viral evolution, resulting from multiple events of recombination between bacterial or phytoplasma plasmids, pre-existing RNA viruses, an even viral satellites.^5,8,31^ Among ssDNA genomes, and in the Virosphere as a whole, Begomovirus is the most prevalent multipartite genus, representing a main contribution to the overall abundance of multipartite species. These successful viruses use a protein called Rep to initiate replication through the rolling circle replication mechanism, which is widely used for plasmid replication in bacteria. It has been hypothesized that a recombination event between an RNA satellite and a phytoplasma plasmid, could have been at the origin of Geminivirus-like viruses.^8,30^ Nanoviridae Reps, unrelated to Begomovirus Reps, are more similar to those found in alpha-satellites or Circoviruses.^5,31^ Therefore, ssDNA viruses are unlikely to stem from a single ancestral virus.

Except for the genus Ourmiavirus, whose RNA-dependent RNA polymerase (RdRp) is related to the Narnaviridae family of RdRps,^32^ the rest of the multipartite RNA families can be rooted in one of the three major superfamilies of RNA viruses infecting eukaryotes (Picornavirus, Alphavirus, and Flavivirus-like).^30^ These facts, together with the diversity of genomes of multipartite viruses (Fig. 2b) suggest that multipartitism could have emerged independently a number of times in evolution.

Although the genomic “pieces” of multipartite viruses are, as far as we know, indistinguishable from those of non-segmented or segmented viruses—those pieces being as old as cellular life itself, and even older for RNA viruses—many plant viruses seem to be recent discoveries of natural selection.^33^ Extant populations of different viral species are only up to centuries old, likely as a result of an evolutionary burst promoted through an intensification of agricultural practices.^34^ Specifically, the radiation of Potyviridae, Luteoviridae, and Sobemoviruses can be traced back to the mid-Holocene and to the beginning of agriculture.^35–37^ Also, there are no evidences of long-term co-evolution between virus and host.^33^ A recent origin of plant viruses, however, does not preclude a broad host range. A single plant virus often infects hosts across plant orders or even classes, suggesting that host switches are frequent despite a lack of obvious co-evolution.^36^ Horizontal gene transfer (HGT) and co-option of molecular functions appear in this context as a more-than-plausible mechanism for the adaptation to new hosts.

## Evolutionary pathways to and from multipartitism

**Fig. 3:**
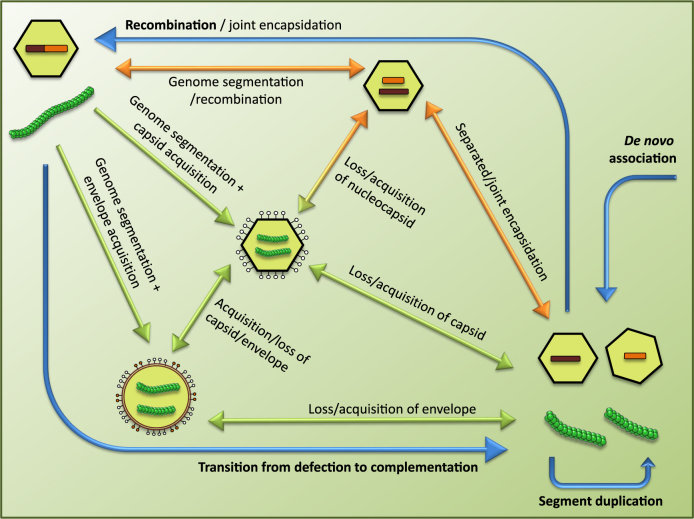
Evolutionary pathways between genomic architectures. Here we represent some possible evolutionary routes relating viruses with non-segmented, segmented and multipartite genomes. Blue arrows correspond to processes that apply to all viruses regardless the capsid type; orange and green arrows correspond to processes that apply to viruses with icosahedral and filamentous/rod-like capsids, respectively. This coarse-grained representation is further discussed in the main text, together with current empirical evidence, if any, and theoretical scenarios supporting the different pathways

The different genome architectures observed in extant viruses might be solutions found after major viral families formed. In general, viruses experience frequent deletion and recombination events during replication, and HGT is common. Actually, homologies that indicate an evolutionary relationship between viruses with different genetic architectures (mono, bi, or tripartite, in early cases) and belonging to separated taxonomical groups have been known for long.^38^ Gene sharing is in all likelihood directly involved in the plasticity observed in viruses at different taxonomic levels. The eventual success of a viral genome structure and architecture results from a highly contingent process. Nevertheless, viral host range does not seem to be conditioned by genome architecture: with the exception of dsDNA viruses, plants are infected by all types of genomes. There are examples of generalists such as the tripartite Cucumber mosaic virus (which infects over 1000 different plant hosts, both mono- and dicotyledons) or the tripartite Tomato spotted wilt tospovirus that infects 360 species from 50 families. However, uniform habitats and vegetative propagation may limit fitness optimization in generalists. Strains of Bean yellow mosaic virus have a limited range to local cultures of domesticated plants and Citrus tristeza virus infections are restricted to a few genera in the *Rutaceae*.^39–41^ Anyhow, most plant viruses are generalists, and less than 10% of plant viruses infect one single host species.^42^ As many as three to four different classes of viruses are often detected in an infected plant,^17,43^ giving plenty of opportunities to explore the joint action of different viral genomes. This behavior could explain in part why multipartitism might have appeared repeatedly in the evolution of plant viruses. Still, possible evolutionary pathways and the specific advantage of multipartitism remain as open questions. In this section we present some ideas in this respect, following the hypothetical pathways depicted in Fig. 3. Evidence to support one or another evolutionary pathway is at present uneven.

### Transitions from non-segmented to multipartite genomes

Defective particles are routinely generated upon replication of viral genomes.^44^ Mutants with changes that preclude their viability in isolation and deletion mutants that lack genes essential to complete the viral cycle can however survive if complemented by viable genomes. Indeed, under conditions of high MOI, defective genomes thrive thanks to the activity in trans of products from viable genomes. In an infection cycle, segmentation might happen through additional mechanisms. Many RNA viruses regulate gene expression through subgenomic RNAs (sgRNA). Encapsidation of sgRNA with loose or non-existent sequence signals is possible.^45,46^ However, as defective genomes, sgRNA particles are frequently lost in presence of the wild type.

There are some in vitro examples of a transition from an originally non-segmented virus to a bipartite one,^47^ and some cases where genetic engineering techniques produced similar outcomes.^48,49^ These facts suggested that an evolutionary transition from a non-segmented virus to a bipartite form should be possible, given an appropriate environment. An experimental demonstration of this possibility was realized with Foot-and-mouth disease virus (FMDV), an animal virus that was subjected to over 200 cell culture passages at a high MOI.^50^ The bipartite, in vitro generated form that spontaneously appeared through evolution of the virus, displaced the wild type in competition under the experimental conditions. Subsequent experiments demonstrated that the superiority of the bipartite form was due to an increased stability of the viral capsid, which translated into an increased particle lifespan.^51^ Finally, when the conditions of propagation were changed to low MOI, the two defective genomes recombined to produce a non-segmented form.^51^ This experiment represents a proof-of-concept that a transition to bipartitism may occur as a result of a change in the ecological context (from low to high MOI in that case).

There is some recent evidence that genome segmentation might be a rare though possible route to multipartitism in virus. This has been suggested for genus Jingmenvirus for which evolutionary relationships with Flavivirus genus (non-segmented) have been established at least for two out of the four segments of the virus—the two other segments are of unknown origin. Flaviviruses infect various arthropods and vertebrates, and are arthropod-borne. Two Jingmenvirus species were described: Jingmen tick virus in ticks, mosquitoes, cattle^52^ and red colobus monkey,^27^ and Guaico culex virus in mosquitoes.^27^ A transition to multipartitism plus a new association to other functional genes could have acted synergistically in the origin of Jingmenvirus.

### Relationship between non-segmented, segmented, and multipartite viral genomes

It could be hypothesized that segmented viruses represent an intermediate state between non-segmented and multipartite genomes, at least for icosahedral viruses (Fig. 3). The rationale behind this possibility relies on the principle of parsimony: a complete genome could break into pieces due to replication errors and those segments could first encapsidate jointly—as it may have happened in the order Mononegavirales which comprises non-segmented and segmented viral families. This situation might favor the subsequent specialization of the segments, deleting overlapping regions and streamlining segment organization. In the final state the segments would be encapsidated in independent viral particles, as may be the case for the family Partitiviridae, which lacks encapsidation signals for the segments. Alternatively, filamentous viruses could have their origin in nucleocapsidic particles formerly enclosed in a complex capsid that at some point released them. Tenuiviruses may have originated through the latter pathway, given the high resemblances with Phlebovirus nucleocapsids.^53^ These similarities between nucleocapsids of segmented Phleboviruses and filamentous capsids are also found in families Closteroviridae and Potyviridae,^54^ which only contain bipartite species. This transition appears as a very plausible way for a recent origin of multipartitism. It may be aided in cases where different or smaller capsids could be horizontally acquired. Furthermore, a new capsid and, in general, the incorporation of novel segments, might grant access to a new collection of possible hosts, with the concomitant increase in the viral population size.

The intermediate segmented state of the former transition might be avoided by viruses with filamentous capsids (Fig. 3), representing an additional example of the transition from non-segmented to multipartite viruses described for Jingmenvirus in the previous section. The origin of filamentous families like Benyviridae and Virgaviridae may include the acquisition of TMV-like capsids in order to infect plants.^54^ Co-encapsidation of segments generated due to replication errors or of sgRNAs is no longer possible, so multipartite encapsidation becomes unavoidable. Alternatively, enveloped animal viruses, with genomes wrapped in nucleocapsids, could be another source for filamentous multipartite viruses.^54^ An example might be Rhabdoviridae, a family of enveloped viruses infecting invertebrates including bipartite genera infecting plants. The transmission from invertebrates to plants, and therefore the adaptation to a new ecological context, might have been concomitant with multipartition.^53^

Reversibility through cooperation of the segments, and genome recombination, is likely a main force to revert to a non-segmented state (Fig. 3). In support of this statement comes the observation that non-segmented species are also found in families containing bi or tripartite viral genera, at odds to what is observed in segmented viral families, which do not simultaneously contain non-segmented or multipartite species.

### Segment duplication

Replication errors and the high numbers of viable and defective genomes simultaneously found within cells might enable mechanisms analogous to gene duplication and subfunctionalization^55^ in viral genomes. Complementation in trans opens the door to the incorporation of new mutations in defective genomes without losing fitness and to, eventually, find new functions. Defective viral genomes can coexist for long in persistent infections, for instance evolving to truly hyper-parasitic forms and eventually causing the extinction of the viral population.^56^ The persistence of defective segments is strongly linked to the frequency of population bottlenecks,^57^ and their presence is rarely observed in vivo. However, it is not unthinkable that the once defective genome might change the overall properties of the initial wild type, allow adaptation to a new ecological niche, and eventually turn out to be essential for the survival of the new, bipartite virus.

Though recent gene duplications in RNA viruses are not abundant, there is evidence of one such event in Benyviridae, a multipartite family.^58^ It cannot be discarded that many duplications are masked due to the rapid evolution of RNA viruses, and that remote paralogs can only be identified through the combined use of non-conventional techniques and manual curation.^59^

Gene duplications are far more frequent in viruses with DNA genomes.^60^ In Begomoviruses,^28,61^ homologies between genes in the same segment have been identified, speaking for duplication events. A mechanism of the kind might have acted to cause the large number of segments of Nanoviruses: some of the parts of this viral family are dispensable in vitro.^29^ Also, there is a significant degree of homology detected between some Nanovirus segments corresponding to regulatory sequences.^62^ Another evidence for the rapid evolution of DNA multipartitism is recombination:^63^ the incorporation of key regulatory sequences that control the addition of foreign genes in Begomoviruses might be instrumental to permit the independent replication of the segments in a short time,^64^ and consequently the expansion to novel ecological niches.^65^

### De novo associations to multipartition

There are many instances in the Virosphere of transient associations of viruses with kin or with subviral agents^17^ that often modify the aetiology of infections. A prominent example are the so-called viral satellites that is, subviral agents that require the assistance of a specific helper virus for its replication or encapsidation.^66^ These associations are ubiquitous in plant infections, and less frequent in animal viruses—with some notable exceptions such as Hepatitis *δ* virus (HDV)^67^ and the genus Dependoparvovirus, in the Parvoviridae family.^68^ A sophisticated kind of satellite-like organism are virophages, which inhibit the replication of their host virus and are typically associated to giant viruses in the Mimiviridae family.^69^ Another interesting class of hyper-parasites are viroid-like satellites consisting on a circular ssRNA dependent on plant viruses for replication and encapsidation, and not coding for any protein. The latter class and HDV are likely related to viroids,^70^ non-coding circular RNAs which have been exclusively described infecting plants.^71^ Some virus-satellite associations^72^ are closely linked to the presence of multipartite genomes. Geminiviridae is the family of plant viruses with the largest number of examples: actually, this family contains many bipartite species but also a large number of species formed by non-segmented viruses that modify their virulence through the action of a satellite.^63^ Additional examples are satellite Tobacco mosaic virus, which worsens the symptoms of the helper virus,^73^ or the generalist CMV, whose association with a fourth non-essential satellite component modifies its virulence depending on the specific satellite^74^ or on the infected host.^75^

Cooperative interactions in mixed infections are also usual for RNA plant viruses. Co-infection of Potyviruses with other species of different families is also related with changes in the aetiology of the infection. Cooperation ultimately leads to interdependence (symbiosis) of two species belonging to different families, as it has been documented for Luteoviruses and Umbraviruses in Groundnut rosette virus or Pea enation mosaic virus. Long-term interactions may end up in speciation, and modular evolution of plant viruses could emerge as a consequence of independent evolutionary histories for individual genes within a genome.^43^

## Gene sharing in the viral world

The power of HGT to cause rapid evolution and adaptation in organisms is difficult to overstate. HGT is ubiquitous and strongly affects the architecture of coding and non-coding parts of genomes. The concept of network genomics^76^ has been put forward to emphasize that the rates of gene gain and loss are comparable to those of point mutations. A recent advance in the quantitative analysis of how relevant gene sharing is to shape current viral diversity has arrived from a study linking genome composition in dsDNA viruses to network theory.^77^ Genes are shared by non-identical subsets of viruses, such that single-gene taxonomy yields incongruent taxonomical trees, because cladograms for genes within a genome do not overlap. Instead, the idea of a network linking genes to the genomes where they are found substitutes the forced time-line of taxonomy by a graphical representation of how genes are shared. The studies carried out to date in this context^77,78^ shed light on possible mechanisms of construction of viral diversity.

Extensions of the former analyses may clarify differences and similarities between the diverse genome architectures found in viruses. At present, we have evidence that gene sharing, and also gene duplication, are processes that take place in multipartite viral genomes, though they do not necessarily represent explanations for multipartitism itself. Still, multipartite viruses might represent one of the most plastic examples of gene sharing. Further data on the age of the associations between parts of such viruses is necessary to find out whether the multipartite strategy has a typical lifespan shorter than that of non-segmented viruses. If they are comparatively more brittle, an advantage of multipartition might lie in its exchange flexibility; if they are equally long-lived, the advantage should be sought in the multipartition strategy itself.

## Advantages and costs

Evidence shows that, regardless the specific situations that permitted the appearance of multipartite viruses, this adaptive strategy yields highly successful plant viruses. A main open question is which are the specific advantages conferred by multipartitism. In that sense, how is multipartitism different from genome segmentation? How is the apparent problem of loss of genomic information in the plant-to-plant transmission solved? The question of the actual advantages of multipartitism in relation to other strategies has received relatively little attention in the virological literature though. However, new and seemingly puzzling experimental results have awoken interest in the problem of the very existence of multipartitism and its adaptive intricacies.^12,14,16^ First, the recently described Jingmenviruses infect mosquitoes, cattle, and non-human primates,^27^ thus broadening the range of hosts of multipartite viruses. This observation suggests that the initial restriction of multipartitism to plant viruses might be a result of a skewed and incomplete sampling.^13^ Second, it has been repeatedly observed that, after an infection cycle, the abundances of different segments of the multipartite genome might vary in orders of magnitude,^14,22,46^ with relative frequencies that seem to be host-specific.^14,79^ The latter observation implies that ensuring maintenance of the genetic information requires a much higher MOI, which will be controlled by the least abundant segment.

There are several possible advantages of multipartition that were put forward early in the literature. One of the most prominent suggestions is the possibility to increase variability through segment shuffling, thus generating hybrid viruses with characteristics from different strains.^80^ Possible applications to genetic engineering were soon suggested.^81^ Advantages in the relationship between parasite and host were also entertained, either related to the replicative machinery of the host^80^ or to the possibility to regulate the relative abundance of genome segments in time and space,^82^ thus controlling cellular resources and host resistance. At the beginning of the 1980 decade only three families (seven genera) of multipartite viruses were known, and all had ssRNA genomes of positive polarity. Multipartitism was implicitly linked to this feature of the virus invoking an increased efficiency in the control of translation.^83^ Exceptions to the observations that led to the advantages just discussed caused soon the dismissal of several hypotheses. The work by Pressing and Reanney^84^ represented an inflexion point in the topic. They argued against increased genetic flexibility, more efficient packaging or more efficient control of translation. Inspired by at the time recent theory that identified high mutation rates as a powerful restriction to the faithful transmission of genetic information in molecular quasispecies,^85^ they devised for the first time a quantitative model of the advantage of bipartition, which would be due to increased copying fidelity of shorter segments.

Pressing and Reanney’s model did not explore how a bipartite form would perform when in competition to its non-segmented analog. In a subsequent model,^86^ conditions for the dominance of the bipartite form considering the MOI and the mutation rate were derived. Also a faster replication rate^87^ was suggested as a putative advantage of shorter genomes, among others.^88^ Those models and the conceptual framework where they were derived originated an interesting controversy in relation to the levels of selection that may affect multipartite viruses (summarized in^89,90^). As of today, experimental evidence of the several possible advantages discussed in the literature is meagre, and the little one that exists does not seem to favor any of the classical hypotheses. The transition to bipartition observed in FMDV^50^ permitted to quantify the advantage of the bipartite form in competition with the non-segmented wild type. Neither a more accurate maintenance of the genetic information nor a faster replication of shorter genomes were detected. Instead, it turned out that viral particles of the bipartite form were more stable, presumably due to a lower pressure exerted on the capsid by shorter RNA segments, allowing for a longer lifetime between infection events.^51^

In any of the scenarios explored, be they qualitative or quantitative, a sufficiently high MOI in transmission was seen as a requirement for persistence. Bona fide advantages of multipartition should compare this strategy with segmentation, and demonstrate that a similar advantage is not to be found in segmented viruses (which enjoy the advantages of shorter genomes without the cost of larger MOI^12^). The precise cost of MOI to maintain a multipartite genome has been analytically worked out^91^ in the scenario of a stochastic deme model.^92^ It has been shown that, under independent propagation of the genome parts, a precise relationship between the gain in average lifetime between infection events due to shorter genomes and the MOI of the propagation exists.^91^ If MOI is not high enough or the gain in stability is too small, the multipartite form will be unable to displace the wild type in competition; otherwise non-segmented and multipartite forms coexist. Analogous relationships are obtained if the advantage relies on the elimination of deleterious mutations or on the replication rate. In any case, the model treats each segment on an equal footing, so it predicts that they will all be equally abundant. This scenario sets a precise limit to the number of segments that can arise under a mechanism of genome segmentation.^91^ For example, a tetrapartite form in competition with its cognate non-segmented counterpart would be fixed only for MOIs above 100–200, well above known typical MOI.^93^ The observation that different segments appear in different amounts puts much more stringent limits on the minimal MOI if they propagate independently. It has been suggested that variable frequency of the segments could however represent a genuine advantage of multipartition by granting additional regulation of gene expression by differences on copy number of the genes.^12,79^ Though the problem of ensuring an MOI able to prevent the dispersion of genetic information remains, empirical knowledge of how multipartite viruses propagate between hosts is very limited. Most plant viruses use vectors to propagate through a broad variety of different interactions.^94^ Despite some evidence suggesting that the number of viral particles transmitted through vectors is too small to fulfill the strict requirements of some multipartite viruses,^95,96^ forms of propagation different from independently drawing the different segments cannot be discarded.^97,98^ High MOI could be facilitated through the formation of complexes containing several heterogeneous particles, through plague-like dynamics of vector populations, or even through movement of the genome parts within the plant tissue—such that the simultaneous presence of all segments in a precise cell before the infection cycle starts would not be required.^99^

## Concluding remarks and prospects

Evolution has been successful in selecting for durable associations between agents that, with independent origins and after a period of competition, eventually found a more stable and robust existence in permanent, cooperative associations. Examples are hypercycles as precursors of metabolism, the transition from separate genes to chromosomes, from individuals to eusocial insects, or a variety of symbiotic associations.^100^ While recombination represents the main driver underlying cooperative association in viruses,^101^ multipartite viruses apparently break that rule. How is that situation reached and maintained? Is multipartitism an adaptive trick, that is a stable strategy where an equilibrium between the cost of high MOI is balanced by other advantages? Or, instead, is it an evolutionary treat, that is the contingent result of transient associations, unstable at long evolutionary times? Both scenarios might be possible in the light of current evidence and, in fact, they do not need to be excluding if multipartitism has appeared several times in evolution: the route to multipartition need not be unique.

Cooperation acquires a broader meaning if ensembles of viral species are portrayed as complex, gene-sharing networks, where cooperation emerges as a distributed property of the ensemble. The Virosphere, and multipartite viruses in particular, might display a systems organization analogous to that observed, e.g., in gene regulatory networks or in cellular metabolism, strongly suggesting the convenience of analysing multipartite viruses with the tools of Systems Biology. Contrary to highly stable associations of genes in chromosomes, gene sharing in multipartite viruses offers an exploding number of combinatorial possibilities that might translate into a myriad of different viral species in short evolutionary time. This constructive principle could be highly relevant in bioengeneering. Could viral segments, once their interactions are uncovered, eventually lead to libraries of viral circuits? A combinatorial use of such libraries might be a first step towards designing or discovering synthetic viral circuits, akin to synthetic gene circuits for cellular genes.^102^ Eventually, this strategy could pave the way to the editing of viral species and to the modification of cellular genomes through viral circuits, with hard-to-foresee implications in bioengineered adaptation. A proof of concept of distributed adaptativity could consist of the elimination of an a priori essential segment in a multipartite genome. Could this procedure be successful as antiviral strategy in the long term? Certainly, the elimination of an essential gene in a virus will cause its extinction in a specific host if virus and host are isolated. But, in presence of the whole network of genes (that is a large pool of viruses none of which infects that host on its own), could the system find cooperative solutions to infect? How fast is the response of the network as a whole? How does that response depend on the size of the gene pool?

Mathematical models will be essential in uncovering the roots of multipartitism. Among others, they might establish under which ecological conditions would the emergence of segmented forms be favored or hindered in competition with non-segmented viral species, or how the presence of suboptimal segments might guarantee survivability. Analysis of gene-sharing networks through quantities defined in complex network theory^103^ should also clarify the topological properties of such networks and how network structure affects their robustness and plasticity, as well as hint at the origins of their overall functionality.

Proteomic studies are revealing a scenario where the evolution of viral and cellular life forms is intimately linked.^54,76^ The origin of extant eukaryotic viruses is traced back to eukaryogenesis and ancient bacteriophages; however, viral species change at a fast pace, such that allocating them in time and establishing their phylogenetic relationships are on-going challenges. When it comes to multipartite viruses, evidence suggests that, as species, they might be just transient opportunistic associations that emerge to permit a fast adaptation to an available ecological niche.^11^ We propose an integrated systems viewpoint where multipartite viral forms need a change in the ecological situation to emerge, be it in the form of a new susceptible host, of a modification in the viral propagation mode, of new genes available in the system or of a host which modifies its availability or mobility, those being challenges that every virus eventually faces. Also, since multipartite viruses are almost restricted to plants, specific properties of the host might condition their lifestyle, as their encapsidation mode or the within-host propagation. In order to disentangle which features of viruses and hosts are essential for the persistence of multipartitism, it would be important to better establish the host range of this strategy. Recent discoveries support that hosts different from plants are not as rare as previously thought. What about sessile marine organisms, and seagrasses in particular? As of today, there are no viruses described infecting marine plants such as *Posidonia oceanica*,^104^ which forms vast meadows of clonal individuals highly reminiscent of monocultures on land. Does *Posidonia* host multipartite viruses? If so, how is the infection transmitted? These are questions that might be instrumental in eventually understanding how multipartitism comes about and persists despite its apparent disadvantages. Admittedly, there might be other ways of alleviating the high MOI requirement that we are not aware of, in which case multipartitism might straight away benefit from advantages such as generation of high diversity and the concomitant fast adaptive response. Perhaps part of the success of multipartitism relies on a dynamical strategy of the type first-come-first-served, being its advantage the fast and opportunistic colonization of new niches.

Plants offer a particularly plastic ground for loose cooperative associations between virus-like agents of different characteristics. A plausible hypothesis is thus that these associations might have turned permanent at the point when conditions drove the partners to an interdependent relationship. An ecological context where infection would be conditional on the joint abilities of a pair of viruses (or of a group of genes) would naturally select for initially multipartite viral forms. We favor the previous scenario as the more plausible mechanistic origin of multipartite viral forms.

Whether associations among fragments is a stable strategy or whether they might evolve to monoparticle viral forms (segmented or not) remains as a further open question.^88^ Actually, there might be restrictions to recombination and single particle encapsidation that either stabilize the multipartite state or at least delay the emergence of a monopartite cognate form—provided it would be a fitter solution. At present, multipartitism seems easier to understand as a transient solution in evolutionary time. The situation might be analogous to islands, whose diversity—according to the theory of island biogeography^105^—is maintained thanks to a constant flux of species from a large reservoir that balances local extinctions. Multipartite viruses might be the dynamic product of a huge and plastic pangenome that is constantly proposing, permitting and sustaining new associations in a complex, changing and diverse global ecology.
